# Magnetic-Core/Gold-Shell Nanoparticles for the Detection of Hydrophobic Chemical Contaminants

**DOI:** 10.3390/nano12081253

**Published:** 2022-04-07

**Authors:** Anna M. Mills, Joseph Strzalka, Andrea Bernat, Qinchun Rao, Daniel T. Hallinan

**Affiliations:** 1 Chemical and Biomedical Engineering Department, Florida A&M University—Florida State University College of Engineering, Tallahassee, FL 32310, USA; amm15s@fsu.edu; 2 Aero-Propulsion, Mechatronics, and Energy Center, Florida State University, Tallahassee, FL 32310, USA; 3 Argonne National Laboratory, X-ray Science Division, Lemont, IL 60439, USA; strzalka@anl.gov; 4 Department of Nutrition and Integrative Physiology, Florida State University, Tallahassee, FL 32306, USA; asb13v@my.fsu.edu (A.B.); qrao@fsu.edu (Q.R.)

**Keywords:** SERS, Raman, grafted nanoparticles, core-shell nanoparticles, hydrophobic, food contamination

## Abstract

Magnetic-core/gold-shell nanoparticles (MAuNPs) are of interest for enabling rapid and portable detection of trace adulterants in complex media. Gold coating provides biocompatibility and facile functionalization, and a magnetic core affords analyte concentration and controlled deposition onto substrates for surface-enhanced Raman spectroscopy. Iron oxide cores were synthesized and coated with gold by reduction of HAuCl_4_ by NH_2_OH. MAuNPs were grafted with polyethylene glycol (PEG) and/or functionalized with 4-mercaptobenzoic acid (4-MBA) and examined using a variety of microscopic, spectroscopic, magnetometric, and scattering techniques. For MAuNPs grafted with both PEG and 4-MBA, the order in which they were grafted impacted not only the graft density of the individual ligands, but also the overall graft density. Significant Raman signal enhancement of the model analyte, 4-MBA, was observed. This enhancement demonstrates the functionality of MAuNPs in direct detection of trace contaminants. The magnetic deposition rate of MAuNPs in chloroform and water was explored. The presence of 4-MBA slowed the mass deposition rate, and it was postulated that the rate disparity originated from differing NP-substrate surface interactions. These findings emphasize the importance of ligand choice in reference to the medium, target analyte, and substrate material, as well as functionalization procedure in the design of similar sensing platforms.

## 1. Introduction

Rapid, sensitive, and reliable detection and quantification of contaminants, such as viruses and bacteria, are of great importance. However, many existing sensing technologies suffer from limited detection ranges and require bulky analytical equipment [1,2]. Moreover, the analytical equipment required is expensive, which limits “household” use. Another obstacle is presented in the wide array of forms in which food can exist [3]. Simple test strips or swabs, common in the medical field, are therefore largely inapplicable to assay of complex food matrices. 

Much of the research that exists in the space of nanotechnology-derived immunodetection techniques focuses on clinical applications, such as sensing target bacteria or viruses. This research has led to some promising data. Wang et al. developed a surface enhanced Raman spectroscopy (SERS)-based lateral flow immunoassay strip using 150 nm Fe_3_O_4_@Ag nanoparticles for the detection of influenza A H1N1 (A/H1N1) and human adenovirus (HAdV). Using this strategy, this group achieved limits of detection of 50 and 10 pfu/mL of H1N1 and HAdV, respectively [4]. Feng et al. combined SERS tags, magnetic supports, and target antigens in a sandwich structure to achieve highly selective and highly sensitive recognition and quantification (in the range of 0.1 ng/mL to 1.0 mg/mL) of human carboxylesterase 1 (hCE1), which is a serum marker for early diagnosis of hepatocellular carcinoma, a common and potentially fatal form of liver cancer. The tags were composed of Ag nanoparticles (AgNPs) functionalized with 4-mercaptobenzoic acid (4-MBA), and the supporting substrates were magnetic nanocomposites composed of Fe_3_O_4_@SiO_2_@AgNPs and functionalized with hCE1 antibodies [5]. Eryilmaz et al. achieved a limit of detection of 3.3 × 10^2^ cfu/mL of Group A Streptococcus pyogenes using Au nanorods with iron oxide cores [1]. The development of a successful sensing platform is largely dependent on the target of interest. These examples demonstrate the wide array of detection platforms developed for solely clinical settings. While bacterial and viral contamination of food products is an ever-present concern, chemical contaminants also pose a myriad of threats to public health [6]. Herein, we explore the effects of a small model target analyte on the efficacy of our sensing platform.

In addition to other vibrational spectrometric techniques, such as mid- and near-infrared (IR) and nuclear magnetic resonance spectroscopy (NMR), SERS has long shown promise in food safety [2,3]. SERS-based immunoassays have been used to detect colorants, drug residues, and a variety of food additives, in addition to more traditional analytes, such as bacteria [1,7,8,9,10,11,12]. SERS has also been used to detect trace amounts of pesticides in foods [6]. One of the difficulties faced in food safety is the complex nature of the assay media [6]. To avoid complicated sample pre- and post-processing steps, ease of retrieval of the NPs from these complex matrices is necessary. By incorporating an iron oxide core, rapid, effective extraction of the composite nanoparticles from complex matrices can be realized [2,5,13]. In fact, magnetic nanoparticles (MNPs) are one of the most popular choices for the detection and capture of food contaminants, due to the possibility of magnetically separating functionalized particles [14]. In other words, after target analytes are immobilized on magnetic particles, they can be rapidly concentrated simply by placing the dispersion in a magnetic field. In addition to the studies mentioned above, Chen et al. achieved detection limits of 10 cfu/mL of E. coli in drinking water using bacteriophage-conjugated magnetic beads [15]. Impressively, contaminated foodstuffs, such as milk, apple juice, lettuce, tomato, and ground beef, have all been processed with good detection performance using a multiplexed MNP immunoassay developed by Kim et al. [16]. Many other sensing platforms have made use of MNPs as immobilization supports instead of magnetically-controlled colloidal particles [17].

Core-shell nanoparticles have potential for sensitive and specific detection of trace analytes present in any complex media, such as food/drink, biological fluids, personal care products, petroleum, and liquid waste streams. Gold, particularly, has long been a choice material for label-free SERS-based immunoassay, because it is biologically inert and exhibits surface plasmon resonance (SPR) [3,18]. Gold is often used in spherical nanoparticle form due to ease of synthesis and SPR enhancement occurring at the appropriate wavelength for detection of organic compounds with Raman spectroscopy [19,20,21,22]. Moreover, the use of gold allows facile functionalization with antibodies specific to the antigen of interest as well as poly(ethylene glycol), which aids in preventing non-specific adsorption, thereby increasing the selectivity of the assay [6].

In the functional design of a SERS-based colloidal sensing platform, interparticle interactions can be dramatically affected by bound functional molecules, presenting critical design challenges. These challenges arise from the complex interplay between interaction potentials (steric, electrostatic, van der Waal’s, depletion, and hydrodynamic) that guide colloidal behavior which require careful control to achieve spatial homogeneity—and therefore reproducibility—of SERS enhancement [23]. While much ongoing research places heavy emphasis on use of gold nanoparticles (AuNPs) in vivo, the use of AuNPs in food safety is relatively recent [24,25,26,27]. Moreover, even fewer studies have documented detection of hydrophobic chemical contaminants, in particular. Hydrophobic molecules possess a low affinity to colloidal Au SERS substrates, and therefore present a significant obstacle in the development of a reliable detection scheme [6]. More research is needed in this area, as this incompatibility has the potential to undermine the selectivity and reproducibility of a sensing platform.

Magnetic-core/gold-shell nanoparticles (MAuNPs) combine the optical properties of gold with the magnetic properties of iron oxide to enable a rapid pre-screening technology. In other words, MAuNPs combine the advantages of facile functionalization and surface plasmon resonance, afforded by the gold shell, with the ability to magnetically separate the particles from complex dispersions, provided by the iron oxide core. MAuNPs are amenable to use in the field via a handheld Raman spectrometer that can detect low-level adulterants, such as those in food, that pose a threat to human health. In this study, iron oxide nanoparticles (MNPs) were coated with gold to create MAuNPs, and their key properties were determined. Samples of MAuNPs grafted with polymer and/or functionalized with a Raman reporter molecule were also examined. Properties of interest include particle size and morphology, colloidal stability, magnetic response, and Raman signal enhancement.

## 2. Materials and Methods

Ethylenediaminetetraacetic acid (EDTA, ≥98%), hydroxylamine hydrochloride (98.0%), hexadecyltrimethylammonium bromide (CTAB, ≥9%), gold(III) chloride trihydrate (≥99.9%), 4-mercaptobenzoic acid (4-MBA, 99%), 4-aminobenzoic acid (4-PABA, ≥99%), and benzyl alcohol (BA) were purchased from MilliporeSigma (Burlington, MA, USA).

### 2.1. Synthesis

#### 2.1.1. MAuNPs

Several synthesis approaches have been investigated over the years, and creation of iron oxide-core/gold-shell nanoparticles has largely required use of inconvenient or hazardous process parameters including extreme temperatures, highly corrosive reagents, and/or extensive reaction times. The former two pose a number of safety hazards. Wet chemistry tactics, such as nano- or microemulsion and reductive deposition, are among the most common methods [28,29,30]. The synthesis method chosen for this work is a modified reductive deposition procedure [25]. This method of iron oxide core followed by gold shell formation was chosen for its convenience. In particular, the time over which the gold reduction reaction takes place is relatively short, and the reaction does not necessitate extreme (high or low) temperatures. In addition, the use of an aqueous solution affords facile gold surface modification through thiol chemistry.

Briefly, MNPs were synthesized by coprecipitation of ferric chloride hexahydrate and iron(II) chloride tetrahydrate in aqueous solution at room temperature. Additional synthesis details can be found in the work of Bernat et al. [31].

By using hydroxylamine hydrochloride as the reducing agent for gold shell synthesis, the following reaction has been proposed: [32]

3 NH_2_OH·HCl +  4 HAuCl_4_·3H_2_O → 3 HNO_2_  +  4 Au^0^  +  19 HCl + 9 H_2_O.

After the reduction reaction takes place, the newly formed gold shell should exhibit an absorbance peak around 530 nm, though the exact wavelength depends upon final particle dimensions and geometry, as described by Mie theory [33,34]. For example, a pure AuNP of 30 nm diameter is predicted to exhibit a peak absorbance at 528 nm, while a MAuNP with a single 10 nm MNP core and a 10 nm Au shell is predicted to have a peak absorbance at 536 nm [35]. In the case of spherical AuNPs, one can use a standard curve and the sample’s peak absorbance to determine the average particle size [36]. More significant SPR shifting occurs for changes in geometry, such as in the case of gold nanorods [37]. Thus, the SPR location in spherical core-shell NPs is not highly sensitive to exact core-shell dimensions. This is especially true when the core material has a much lower molar attenuation coefficient than the shell [38]. More important design considerations are ensuring a thin enough gold shell to maintain magnetic response but a thick enough gold shell to ensure complete coverage. Incomplete coverage can cause adverse ligand grafting effects and even corrosion.

The gold coating process was executed as outlined by Tamer et al. with minor modifications [25]. Approximately 10 mg of as-synthesized MNPs were combined with deionized (DI) water, sonicated for 30 s, and centrifuged for 10 min at 3000 rpm. This washing procedure was performed three times. Centrifugation of unstabilized MNPs is preferable to magnetic separation to avoid irreversible agglomeration. The resulting precipitate was re-dispersed in 5 mL DI water by sonicating for 5 min. Then, 5 mL 0.27 M EDTA, prepared in 1 M NaOH, was added. An overhead stirrer was used to agitate the reaction mixture from above, while an ultrasonic bath provided sonication from below. The solution was agitated for 5 min and then centrifuged for 30 min at 3200 rpm. After the supernatant was discarded, DI water was added and the solution was sonicated for 30 s, then centrifuged for 15 min at 3200 rpm. This washing procedure was performed three times. The EDTA-immobilized MNPs (EDTA-MNPs) were transferred from the centrifuge tube to a round-bottom flask. Subsequently, 7 mL of 0.1 M CTAB, 3 mL of 0.01 M HAuCl_4_, and 0.3 mL of 1 M NaOH were added to the EDTA-MNP dispersion. The aqueous mixture was stirred vigorously for 30 s. Finally, 150 mg hydroxylamine hydrochloride was added, and the mixture was stirred vigorously for 3 min. The resulting ruby-colored solution remained in the flask for 24 h to allow acidic digestion of uncoated MNPs. Upon completion, the MAuNP dispersion was centrifuged at 3000 rpm for 5 min, the supernatant was discarded, and DI water added. Centrifugation is preferable at this stage to preferentially collect the larger MAuNP particles and discard smaller MNPs in the supernatant, if any remain. This washing procedure was performed three times. The final concentration of the MAuNPs was about 4.37 mg/mL, as determined by UV-vis (molar attenuation coefficient, ε = 3.36 × 10^9^ M^−1^ cm^−^^1^) [39]. Detailed calculations are provided in the Supplementary Materials.

#### 2.1.2. Functionalization

Methoxy poly(ethylene glycol) thiol (mPEG-SH, 5 kg/mol, Laysan Bio., Arab, AL, USA) was used in this study, because it is a common ligand used to stabilize MAuNPs and prevent nonspecific adsorption. Aqueous solutions of mPEG-SH were prepared in varying concentrations (0.2, 2, and 20 mM). For each sample, 10 µL MAuNPs were added with 1 mL mPEG-SH solution and magnetically stirred overnight. These functionalized MAuNPs, denoted PEG-MAuNPs, were then extracted and added with DI water in a centrifugal filter unit (Amicon Ultra, 50,000 g/mol molecular-weight cutoff). Samples were centrifuged for 5 min at 3000 rpm. The rinsing procedure was performed three times to remove uncoordinated mPEG-SH.

Motivated by the desire to focus on hydrophobic detection, the Raman reporter molecule chosen for this study was 4-mercaptobenzoic acid (4-MBA) on account of its poor solubility in water [40]. To functionalize the MAuNPs with 4-MBA, aqueous MAuNPs were dried in air (10 μL solution for Raman, approximately 2.3 mg for magnetophoretic separation) and re-dispersed in chloroform (10 μL for Raman, 50 μL for magnetophoretic separation). The re-dispersed particles were added with the specified reporter concentration (0.1, 1, or 10 mM for Raman, 0.1 mM for magnetophoretic separation), also prepared in chloroform (1 mL for Raman, 5 mL for magnetophoretic separation). The sample was then sonicated in an ultrasonic bath for one minute and placed in the dark to incubate. The incubation duration was 24 h for each sample. After incubation of MAuNPs in 0.1, 1, or 10 mM 4-MBA solution, samples were centrifugally rinsed (3000 rpm, three times in 5 mL chloroform), unless otherwise specified.

### 2.2. Experimental Techniques

Dynamic light scattering (DLS) was performed using a Nicomp 380 Particle Sizing System at a fixed detector angle of 90°. All samples reported were prepared in concentrations between 0.1 and 1 mg/mL in water and sonicated for 30 s immediately preceding measurement, unless otherwise noted.

Transmission electron microscopy (TEM), elemental mapping, and energy dispersive X-ray spectroscopy (EDS) were performed using a JEM-ARM200cF at 200kV, which was equipped with Oxford Aztec EDS SDD detector at the National High Magnetic Field Laboratory in Tallahassee, FL. TEM samples were prepared by iterative drop-casting onto holey carbon-coated copper grids (Electron Microscopy Sciences). As-synthesized MAuNPs were diluted 10% by volume in DI water and iteratively cast 3 to 5 times in 4 μL increments. MNP samples were prepared in a similar manner. Samples were allowed to dry for at least 10 min before examination. Particle sizing was performed by processing micrographs in MATLAB, whereas Gatan DigitalMicrograph software was used to probe lattice fringe spacing of high resolution TEM (HRTEM) images via Fast Fourier transform (FFT). Elemental mapping was acquired with a probe size of 0.12 nm in scanning transmission electron microscopy mode.

To assess the average diameter of the MAuNPs, grazing-incidence small-angle X-ray scattering (GISAXS) was performed at the 8-ID-E beamline under vacuum at room temperature at Argonne National Laboratory in Lemont, IL. Samples were functionalized with PEG (0, 0.2, 2, or 20 mM) and/or 4-MBA (0, 0.1, or 1 mM), transferred from water to chloroform, and drop-cast onto silicon substrates in a technique described by Yang et al. [22]. All GISAXS data were collected on a Pilatus 1M detector positioned 2185 mm from the sample using an incident angle of 0.14° and photon energy 10.9 keV. Exposure duration was 10 s. 1D linecuts were generated from 2D scattering profiles by integrating intensity of a thin, horizontal slice of the detector data from 0.0087–0.2082 Å^−1^ directly above the Yoneda band.

A physical property measurement system (PPMS) (16 T, Quantum Design) equipped with a vibrating sample magnetometer (VSM) was used to characterize the magnetic response of the MNPs. Approximately 0.1 mg MNPs were dispersed in a cylindrical epoxy mold and examined at room temperature (298 K), using a frequency of 43 Hz, amplitude of 0.25 mm, and averaging time of 0.75 s. The magnetic field was swept between ± 20,000 Oe at a rate of 77 Oe/s. In the interest of preserving data comprehensiveness, VSM was not used to characterize the MAuNPs. The methods utilized herein would have required a significant fraction of the MAuNPs synthesized in a single batch to guarantee data quality.

^1^H NMR spectra were collected with a 600 MHz spectrometer (Bruker) using water suppression. For each sample, 64 spectra were collected and summed. A detailed description of the NMR sample preparation procedure is provided in the Supplementary Materials.

Silicon substrates for Raman measurements were cleaned with piranha solution, rinsed with DI water, and dried. (4-MBA)-MAuNPs were drop cast from chloroform onto silicon wafers. Control samples were prepared by direct drop-casting of Raman reporter solutions in chloroform (3 μL of 0.1, 1, or 10 mM deposited with a micropipette) onto a Si substrate. Raman spectra were collected with a confocal Raman microscope (Renishaw InVia) using a 785 nm diode laser source (5 mW power at sample position) and an exposure time of 10 s. The spot diameter was approximately 2 × 10^−3^ mm. Spectra were analyzed with Renishaw’s WiRE 3.4 software. 

Magnetophoretic separation experiments were conducted using one-inch NdFeB, N52 permanent magnets (K&J Magnetics, DX0X0-N52). Magnetophoresis of MAuNPs was studied in water and in chloroform. As-synthesized MAuNPs were dispersed in water at 0.02 mg/mL. As-synthesized MAuNPs were also transferred to chloroform at 0.02 mg/mL. Deposition of the as-synthesized MAuNPs in chloroform was studied with and without a magnet, the latter serving as control. Two sets of 4-MBA functionalized MAuNPs were studied: one set was rinsed as described above and dispersed in 30 mL chloroform, while the other set was separated directly from the incubation solution after 24 h. In both cases, 30 mL of (4-MBA)-MAuNP dispersion was distributed evenly among three vials, at the bottom of which laid a radially-centered, pre-massed silicon wafer. The vials were each immediately placed directly on top of a permanent magnet, where they remained for 10, 100, or 1000 min. At the end of the deposition experiment each solution was carefully decanted while remaining positioned on its magnet. The vials were then removed from the magnets and dried in air. The wafers were again massed to determine the deposited mass of MAuNPs. 

## 3. Results and Discussion

**Figure 1:**
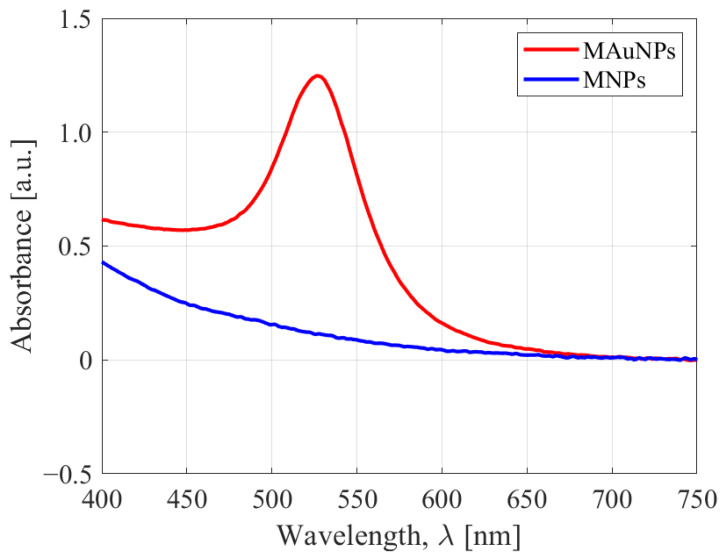
UV−vis spectra of MNPs (blue) and MAuNPs (red). The MAuNP absorbance band is centered around 529 nm.

The UV-vis spectra of uncoated and coated particles are compared in Figure 1. The MAuNPs clearly exhibit an absorbance peak at about 529 nm, while the peak is entirely absent from the spectrum of MNPs. This indicates that the gold coating process was successful.

DLS measurements indicate that the average hydrodynamic diameter for the dominant population of MNPs is 57.8 ± 7.4 nm and that of MAuNPs is 71.7 ± 12.5 nm. The particle volume fraction of the optically transparent DLS samples (<10^−6^) was assumed to be sufficiently low, such that the particles were non-interacting. Despite this, the diameter values are significantly larger than those from TEM measurements. The larger hydrodynamic diameter from DLS can be attributed to the presence of secondary, weakly flocculated particles or to large solvation sheaths. As is common in DLS, stabilizing surface charges or ligands can artificially inflate the measured particle size (i.e., hydrodynamic diameter) due to the differences in zeta potential [23]. The MNPs were stored in an alkaline solution (pH ≈ 11) in order to ensure surface coverage by EDTA. Similarly, the MAuNPs were stored in an acidic solution (pH ≈ 4) in order to promote surface functionalization by CTAB [41]. Upon preparing particles for DLS by rinsing with DI water, the coordination between ligands and particle surfaces was destabilized, leading to weak flocculation of the EDTA-MNPs and CTAB-MAuNPs. In this way, the presence of EDTA appeared to mitigate irreversible aggregation, but facilitate reversible agglomeration, of MNPs in aqueous solution. In order to investigate agglomeration of EDTA-MNPs, particle size distribution was collected periodically using DLS. Over time, the appearance of larger secondary particles was observed. Upon removing the sample and sonicating for another 30 s, evidence of larger secondary particles vanished before reemerging slowly over the course of a few minutes (see Figure S1).

**Figure 2:**
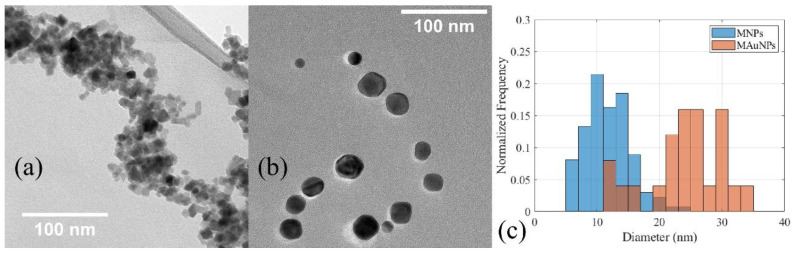
Representative TEM micrographs of (**a**) MNPs, (**b**) MAuNPs, and (**c**) the distribution of NP sizes as measured from TEM micrographs.

Since DLS measurements are sensitive to interparticle forces, TEM was also used to assess the particle diameters [42]. Representative bright field micrographs are shown in Figure 2. A MATLAB script calculated the size of individual particles based on user-generated circles that were superimposed around each particle. Using this script, the MNPs were found to be 10.8 ± 1.9 nm in diameter, and the MAuNPs were found to be 27.2 ± 12.4 nm in diameter. Assuming each MAuNP has a single MNP core, the nominal gold shell thickness (Δt) was approximately 8.2 ± 6.2 nm, as calculated by Equation (1), where D_MAuNP_ is the average diameter of the MAuNPs, and D_MNP_ is the average diameter of the cores.

(1) $$\Delta t=\frac{D_{\mathrm{MAuNP}}-D_{\mathrm{MNP}}}{2}$$

Over 100 particles each were examined in the size determination of MNPs and MAuNPs. Additionally, after coating, TEM showed evidence of uncoated MNPs, so EDS and FFT were also performed to identify and discern between coated and uncoated NP populations, as well as provide invaluable information on the general composition and morphology of individual particles.

**Figure 3:**
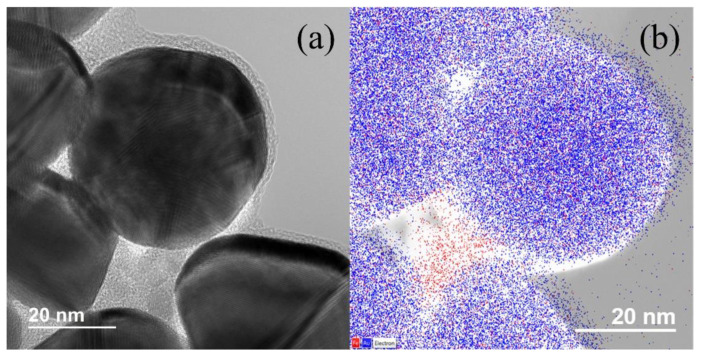
(**a**) HRTEM micrograph of MAuNPs (larger darker particles) and uncoated MNPs (smaller lighter particles). Gold and iron oxide lattice fringes are apparent. (**b**) EDS element map performed on a similar region of the same sample depicting Fe (red) and Au (blue).

Using EDS, the two-dimensional distribution of both iron and gold were sampled and mapped. Figure 3 depicts an example of such a map, created from an image of the crude dispersion. Iron is shown in red, while gold is shown in blue. Although there is evidence of sample drift during acquisition, there is clearly a strong presence of both gold and iron within the larger particles, and a clear absence of gold in the intermediary areas containing small particles. The percentage of MNPs successfully coated in Au was estimated by comparing the theoretical shell thickness with the average shell thickness observed in TEM. The model to calculate the theoretical shell thickness assumed that the HAuCl_4_ reduction reaction occurred both completely and stoichiometrically, and that each MNP was coated uniformly by a gold coating with the same density as bulk gold. With these assumptions, the shell thickness was predicted to be ~1.5 nm, which is thinner than what was calculated from TEM measurements (~8.2 nm). By assuming the total volume of Au reduced contributed to a uniform coating thickness of 8.2 nm on the MNPs, the number of coated MNPs was estimated to be 7% of the total number of MNPs. Qualitatively, the proportion of residual MNPs observed in TEM was much lower than this estimate, but the cause is unclear—some amount of MNPs could be lost during synthesis, either in the rinsing steps or in the final 24-h “resting” step, during which uncoated MNPs are subjected to acidic conditions and dissolved. Given the broad areal distribution of iron in the EDS element map, it is also possible there exist multiple cores within a single MAuNP.

**Figure 4:**
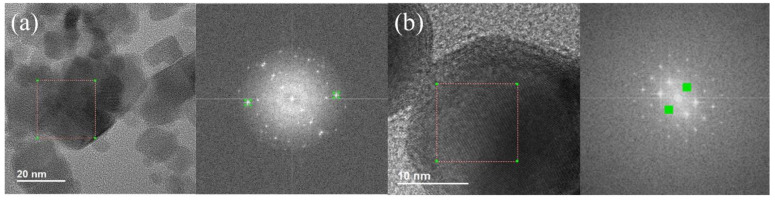
TEM images with selected area for FFT denoted by the red dashed box and the corresponding diffraction patterns of (**a**) MNPs and (**b**) MAuNPs.

FFT was performed to corroborate the EDS findings. Example micrographs and their FFT diffraction patterns are given in Figure 4 for the MNPs (left) and the MAuNPs (right). It was found that the most prominent distances exhibited by MAuNPs were 0.236 ± 0.016 nm and 0.205 ± 0.019 nm, which are consistent with Au(111) and Au(200), respectively [43]. A similar analysis was completed for the MNPs. They exhibited a wider array of spacings, all falling within reasonable range of Fe_3_O_4_(511), (311), (222), (400) or Fe_2_O_3_(220), (222) [44]. Interestingly, MNPs that remained uncoated after they underwent the gold-coating procedure strictly exhibited spacings consistent with Fe_2_O_3_(400). These findings further support the EDS data. Some MNPs remain in the MAuNP solution, which may indicate the need to incorporate a purification step beyond centrifugal filtration, if residual MNPs pose a problem for a particular application. 

**Figure 5:**
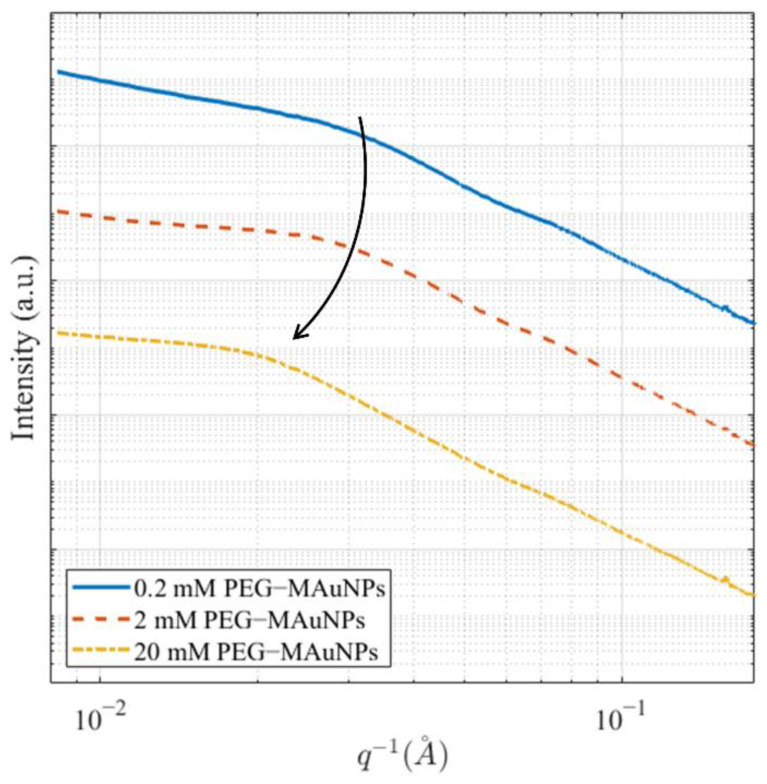
Comparison of raw scattering data of 0.2, 2, and 20 mM PEG-MAuNPs. The arrow depicts the shift in the scattering peak with increasing PEG concentration. Data sets are offset for clarity.

One-dimensional projections of two-dimensional GISAXS data are shown in Figure S2. These scattering patterns were analyzed with SASView. The raw scattering data for the 0.2 mM, 2 mM, and 20 mM PEG-MAuNPs are shown in Figure 5. One can interpret the shoulder of these curves as the most probable pair distance, or center-to-center distance [45]. To better interpret the data, real-space distance distribution functions, *P*(*r*), were calculated with SASView by fitting the raw scattering intensity in reciprocal space and assuming the local monodisperse approximation holds [46]. The center-to-center distance of the PEG-MAuNPs, in increasing order of concentration, were found to be 8.3 ± 4.8 nm, 7.4 ± 4.6 nm, and 9.9 ± 5.0 nm, respectively. These are shown in Table 1 for all samples.

Examining Figure 5, the 20 mM PEG-MAuNP sample exhibited a smaller *q*-value at the shoulder as compared to the 0.2 mM PEG-MAuNP sample. This indicates an increased effective particle size. However, the standard deviations reported in Table 1 suggest that the difference is not statistically significant. In any case, the non-monotonic trend in most probable pair distances is unexpected. Densely-grafted colloidal particles sometimes exhibit net attractive interactions, depending on the nature of the polymeric species and suspending medium. This phenomenon is well-documented for polymer melts [47]. On the other hand, sparsely-grafted colloidal particles may also show net attractive interparticle interactions, since the steric repulsive forces provided by the polymer chains are inconsistently distributed on the particle surface [48]. Furthermore, once cast onto substrates, particles often aggregate due to drying effects [49]. In this experiment, pair distance was not explicitly controlled; however, simple solvent casting has been used for particle pair distance regulation [50]. Due to the complexity of possible interactions and uncertainty in GISAXS results, scanning electron microscopy (SEM) was used to qualitatively examine the structure of drop-cast MAuNPs and 0.2 mM PEG-MAuNPs. Micrographs are provided in Supplementary Materials Figure S3. Each of the drop-cast samples formed cluster agglomerates separated by bare substrate, indicating that drying effects likely played a significant role in the final positioning of individual MAuNPs.

The effects of functionalization sequence were also explored. A total of 2 mM PEG-MAuNPs were incubated in either a 0.1 mM 4-MBA solution or a 1 mM 4-MBA solution. Two other MAuNP samples were incubated, first with 4-MBA (again, one in each concentration), and subsequently subjected to grafting in 2 mM PEG solution. As shown in Table 1, the samples first grafted with PEG exhibited smaller separation distances than those that were first incubated with 4-MBA, but the difference is statistically insignificant. Interestingly, in comparison to the 2 mM PEG-MAuNPs, samples incubated with both 4-MBA and PEG exhibited consistently larger particle spacings, regardless of functionalization sequence; however, 4-MBA alone was not found to increase interparticle spacing. This effect was investigated further with NMR.

**Figure 6:**
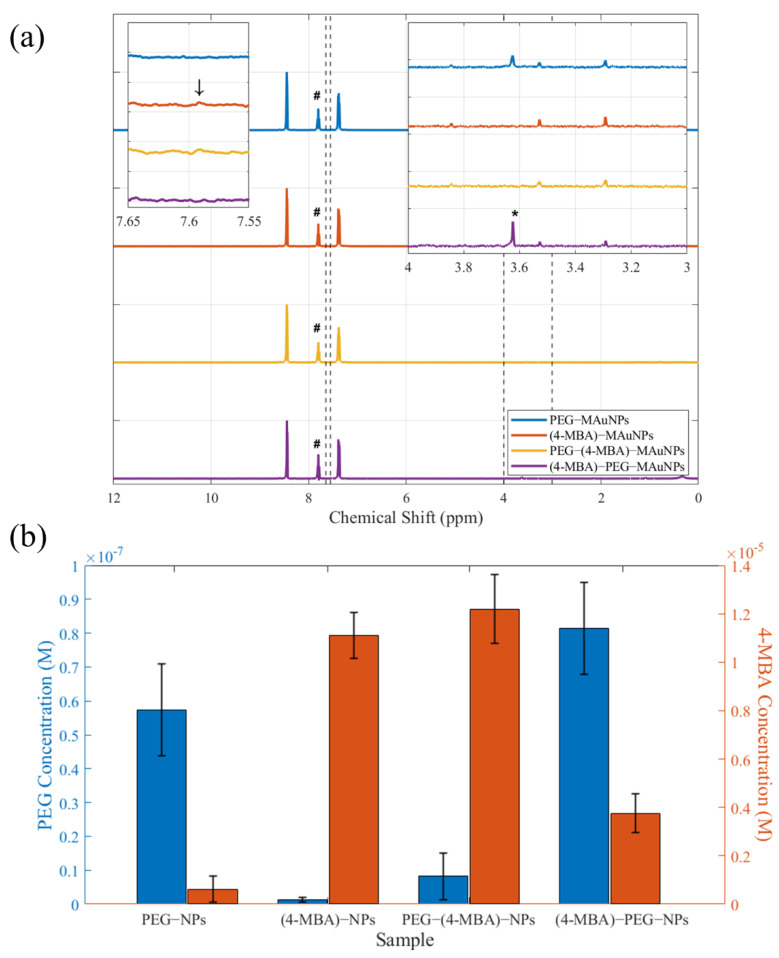
(**a**) NMR spectra of PEG-MAuNPs (blue), (4-MBA)-MAuNPs (red), and PEG-(4-MBA)-MAuNPs (yellow). All samples were incubated in 2 mM mPEG-SH solution and/or 0.1 mM 4-MBA solution. Insets ranges are denoted by vertical dashed lines. (**b**) Calculated concentrations of PEG (blue) and 4-MBA (orange) from spectra in (**a**) using pyridine reference peak denoted by #.

The NMR chemical shifts are provided for 2 mM-PEG-MAuNPs (blue), 0.1 mM-(4-MBA)-MAuNPs (red), a sample of MAuNPs that was first grafted with 2 mM PEG solution and subsequently functionalized with 0.1 mM 4-MBA (yellow), and MAuNPs first functionalized with 0.1 mM 4-MBA and then grafted with 2 mM PEG (purple) in Figure 6a. The three prominent peaks between 7.2 and 8.6 ppm are attributed to the pyridine reference. In the inset on the left, the weak 4-MBA peak at 7.59 ppm is attributed to the protons on the aromatic ring and is denoted by an arrow. In the inset on the right, the peak at 3.29 ppm is attributed to EDTA, the peak at 3.53 ppm is attributed to CTAB, and the peak at 3.63 is attributed to the methyl end-group of mPEG-SH and is denoted by an “*” [51]. It is worth noting that the PEG peak is much stronger and narrower than the 4-MBA peak. The PEG molecule grafts to the MAuNPs via the thiol group on the opposite end of the 5 kg/mol chain. Thus, grafting has much less impact on the mobility of the PEG methyl group than that of the 4-MBA aromatic protons. Reduced mobility and the presence of iron particles are known to cause NMR peak broadening [52]. The peak marked with a “#” is attributed to the α-proton in pyridine [50]. Using this peak as reference, PEG and 4-MBA concentrations were determined. The results of the peak integrations are provided in Figure 6b, where the PEG concentration is shown on the left axis (in blue) and the 4-MBA concentration is shown on the right axis (in orange). In addition, graft density (σ) was estimated using Equation (2), where x is the number of moles of ligand, N_A_ is Avogadro’s number, and N is the number of particles. Values are shown in Table 2. Error values shown were calculated using the standard deviation of the NMR data and the TEM D_MAuNP_ data.

(2) $$\sigma =\frac{x\cdot N_{A}}{N\cdot \left(D_{\mathrm{MAuNP}}^{2}\pi \right)}$$

The PEG concentrations measured using NMR tell a similar story to the GISAXS data; samples first grafted with 4-MBA showed higher final PEG concentrations than those grafted with 4-MBA second. As shown in Table 1, particle spacing was greater in samples first grafted with 4-MBA than in those grafted with 4-MBA second. The latter samples would, therefore, have diminished steric repulsive potential and be less able to inhibit interparticle attractions, while the former samples would be more able to provide a sufficient repulsive potential. NMR results also indicate that samples first grafted with PEG showed lower final PEG concentrations after functionalization with 4-MBA than samples not exposed to 4-MBA. Thus, 4-MBA is clearly displacing some PEG ligands. This phenomenon we believe to be a result of ligand exchange, as concluded in a study by Smith et al. [53]. CTAB is being exchanged for PEG or 4-MBA in the first NP modification step, but backfilling can also occur. Backfilling is a nuanced process in which new ligands fill in the gaps left between existing ligands on the NP surface. In the study performed by Smith et al., NMR was used to probe the ability of a short thiol-terminated ligand (11-mercaptoundecanoic acid, MUA) to displace a long thiol-terminated ligand (1 kDa mPEG-SH), and vice versa, on 30 nm AuNPs. They found that self-assembled monolayers (SAMs) composed of PEG, when exposed to an excess of MUA, had a higher exchange efficiency (95%) than SAMs made of MUA and exposed to an excess of PEG (< 2%). This effect is due to the enhanced ability of a small molecule to locate and make use of macromolecular SAM packing defects, in comparison to the situation in which the roles are reversed. Our results agree with the general trends seen in this study. In fact, samples exposed to PEG, then 4-MBA, have higher total ligand concentration than those exposed only to 4-MBA. Similarly, samples exposed to 4-MBA, then PEG, have higher total concentration than those exposed only to PEG. Thus, in addition to ligand exchange, backfilling also must be occurring. This would also explain why samples exposed to both PEG and 4-MBA appear to have larger probable pair distances than samples without 4-MBA.

**Figure 7:**
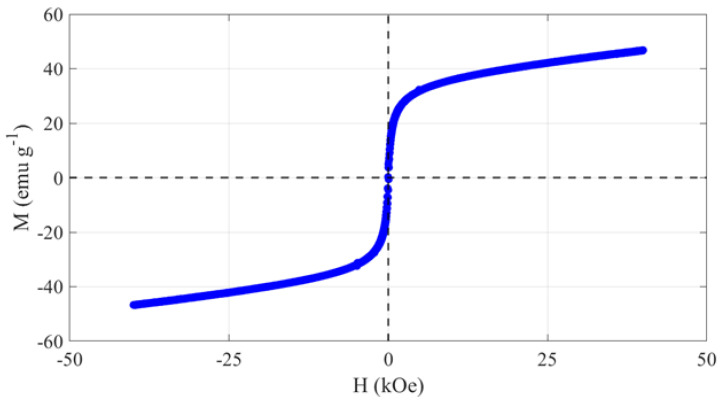
Magnetic hysteresis loop of the MNPs from VSM measurements.

**Figure 8:**
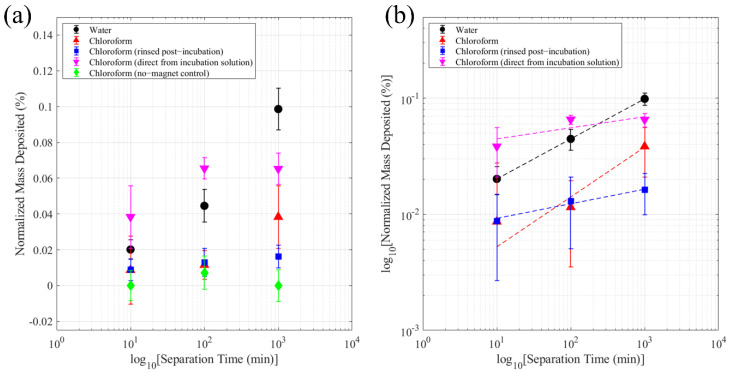
Mass values were normalized by the mass of the clean substrate as follows: normalized mass deposited =(measured mass – substrate mass)/substrate mass ×100%. (**a**) Percentage mass deposited is presented as a function of time and separation medium. (**b**) Log-log plot of percentage mass deposited versus time. Dashed lines represent power law fits.

VSM measurements (Figure 7) show the room temperature magnetic hysteresis curve of the MNPs. As is characteristic of superparamagnetic materials, they exhibit limited hysteresis. The saturation magnetization (M_s_) was found to be around 42 emu/g. These magnetic characteristics of the particles suggest they are suitable for separation. To confirm, magnetophoretic separation experiments were conducted. The separation efficacy was evaluated on a mass basis and contrasted with a control trial without a magnet. As shown in Figure 8a, after 1000 min of separation without a magnet, the MAuNPs did not sediment appreciably from solution, emphasizing the role of magnetic attraction in particle deposition. The sample dispersions exhibited remarkable stability at this concentration, as evidenced by the no-magnet control sample. With the exception of the control, the same data from Figure 8a is displayed on a log-log plot in Figure 8b. Lines represent power law fits ($m=At^{b}$). The fit results are shown in Table 3. From this data, a trend appears—samples separated from pure DI water or chloroform (black circles and red triangles, respectively), without having ever come into contact with 4-MBA, exhibit larger slopes, corresponding to faster rates of mass deposition than the (4-MBA)-MAuNPs (pink upside-down triangles and blue squares). Regardless of whether the (4-MBA)-MAuNPs were rinsed or deposited directly from 4-MBA/chloroform incubation solution, the rate of mass deposition was slower than it was in the absence of 4-MBA. This is thought to be due to unfavorable interactions between (4-MBA)-MAuNPs and the substrate. Prior to the experiment, the Si substrates were cleaned using piranha solution, which is known to result in a generally hydrophilic surface [54]. Since 4-MBA is a hydrophobic molecule, unfavorable NP-substrate interactions are postulated to be the primary source of mass deposition rate reduction.

An interesting case is that of the MAuNPs in chloroform medium. The power law exponent (b≈0.5) qualifies the system as being in the “diffusive” (as opposed to the “ballistic”) limit of the magnetophoretic constitutive relationship proposed by Ayansiji et al. [55]. The total flux, when considering an ideal solution of charge-neutral, non-interacting nanoparticles exposed to a stationary magnetic field is provided by Equation (3)

(3) $$\vec{N}=\left(\frac{D_{m}}{D_{e}}-1\right)\vec{\nabla }c+\frac{{\vec{v}}_{m}}{D_{e}}c,$$

where $D_{m}$ is the magnetic diffusion coefficient of the NPs, $D_{e}$ is the effective diffusion coefficient of the NPs, ${\vec{v}}_{m}$ is the magnetic velocity of the NPs, and c is the NP concentration. Given the strength and proximity of the N52 permanent magnet, the effects of magnetic convection are expected to be negligible due to the approximate uniformity of the magnetic field gradient over the separation volume. In other words, the magnetic velocity is proportional to the gradient of the magnetic field, which is approximately zero. Thus, diffusive deposition is expected in our set-up. When $\frac{D_{m}}{D_{e}}<1$, deposition does not occur because concentration-gradient driven diffusion dominates over magnetic diffusion. On the other hand, when $\frac{D_{m}}{D_{e}}\geq \;1$, deposition can occur. When $\frac{D_{m}}{D_{e}}\sim 1$, deposition will be strongly affected by the boundary condition. In the absence of 4-MBA, favorable surface-particle interactions impede desorption of deposited particles. Whereas, in the presence of 4-MBA, surface-particle interactions are unfavorable, such that deposited particle can desorb and diffuse away, which is thought to explain the much slower deposition rate in the presence of 4-MBA.

**Figure 9:**
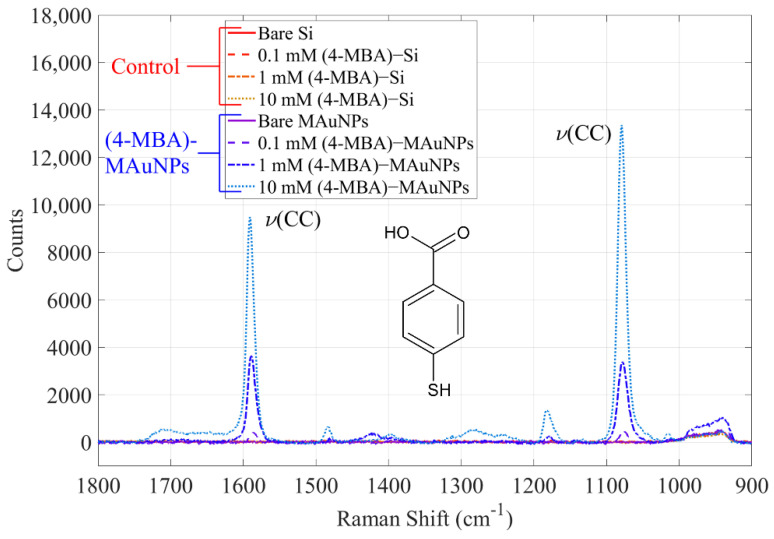
Raman spectra demonstrating the 4-MBA signal enhancement afforded by (4-MBA)-MAuNPs (blue) over control silicon wafers and unfunctionalized MAuNPs (red).

To assess the detection capability of functionalized MAuNPs, Raman spectra were collected. The results of this study are shown in Figure 9. The control samples consisted of bare silicon wafers coated with 4-MBA and are shown in varying shades of red. In contrast, the MAuNPs are shown in shades of blue or purple. Raman enhancement was characterized on the basis of their enhancement factor (EF) as calculated using Equation (4)

(4) $$\mathrm{EF}={\left(\frac{I_{\mathrm{SERS}}}{I_{0}}\right)}^{2},$$

where I_SERS_ is the integrated intensity of the enhanced Raman signal, and I_0_ is the integrated intensity of the Raman signal from a silicon substrate drop-coated with the same 4-MBA concentration as the MAuNP incubation solution.

**Figure 10:**
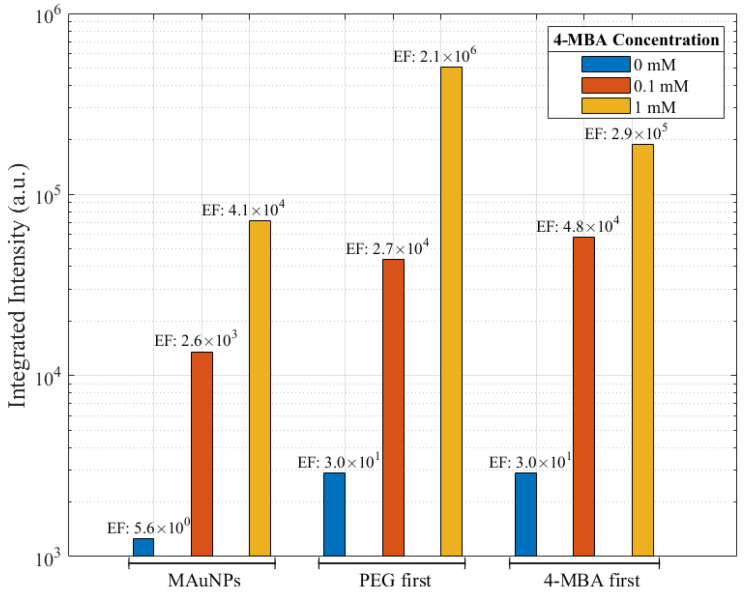
Comparison of integrated intensity of SERS spectra for samples incubated with 0, 0.1, or 1 mM 4-MBA. Values represent integrations from 1028 to 1128 cm^−1^. Calculated Raman enhancement factors (EFs) are transcribed above each of their respective data bars. Samples indicated as either “PEG first” or “4-MBA first” were grafted with 2 mM PEG, and the 0 mM 4-MBA sample was only grafted with PEG. It is shown for reference.

The MAuNPs show significant signal enhancement at Raman shifts of around 1079 cm^−1^, which is attributable to C-C bond vibrations (ring-breathing mode) characteristic of 4-MBA [56]. The 10 mM-(4-MBA)-MAuNPs achieved an EF of 8 × 10^9^. Integrated peak intensity and EFs of samples incubated with 0, 0.1, or 1 mM 4-MBA are shown in Figure 10. The 1 mM 4-MBA samples exhibited an order of magnitude higher EF for the particles functionalized with 4-MBA second as compared to those functionalized with 4-MBA first. For 0.1 mM 4-MBA, the functionalization sequence did not show a clear EF trend. At both concentrations, the PEG-(4-MBA)-MAuNPs, or “PEG first”, and the (4-MBA)-PEG-MAuNPs, or “4-MBA first”, achieved greater Raman enhancement in comparison to the (4-MBA)-MAuNPs, or “MAuNPs”, that were never exposed to PEG. All enhancement factors were significantly greater than the baseline signal, which is given by EFs from 0 mM 4-MBA measurements on as-synthesized particles (MAuNPs in Figure 10) and particles only grafted with 2 mM PEG (PEG first and 4-MBA first in Figure 10).

The EFs are not in agreement with the 4-MBA concentrations estimated with NMR, as NMR suggested the PEG-(0.1 mM-4-MBA)-MAuNPs possessed the highest concentration of 4-MBA, followed by (0.1 mM-4-MBA)-MAuNPs and (0.1 mM-4-MBA)-PEG-MAuNPs, respectively. The discrepancy may have arisen from inhomogeneity of the SERS samples, or could be due to the extremely weak 4-MBA NMR signal. Comparisons with the literature indicate the EFs span the range typical for AuNPs (10^3–^10^9^), with the 0.1 mM-(4-MBA)-MAuNPs on the lower end and 10 mM-(4-MBA)-MAuNPs on the upper end [21,57,58]. Another study including higher 4-MBA incubation solution concentrations might elucidate the differing trends seen in the NMR and SERS data.

The versatility of the detection platform was demonstrated with two other analytes: PABA and BA. The SERS spectra of these analytes is shown in Figure S4. Despite the similarity of their chemical structures, the Raman spectra have unique fingerprints, such that they are readily differentiated. The EF range agreement with AuNP-focused literature is a promising aspect of the system investigated in this study. Reducing the amount of gold mass in a NP without compromising the Raman enhancement would reduce the overall cost of the system, and enabling magnetic separation is convenient for detection in complex media. SERS of magnetic core-shell nanoparticles is thus a powerful technique for sensitive detection, even of hydrophobic analytes. In addition, MAuNP dispersions (~0.5 mg/mL) remained visibly stable over the course of about 3 months. On can envision a versatile platform that combines this approach with principle component analysis, machine learning, or even artificial intelligence to detect specific analytes in complex mixtures, such as food matrices.

## 4. Conclusions

In this study, MNPs were coated with gold using a reductive deposition procedure to create MAuNPs, and their key properties determined. Samples of MAuNPs grafted with polymer and/or functionalized with 4-MBA were also examined. In (4-MBA)-PEG-combined samples, it was found that 4-MBA increased interparticle spacing, regardless of functionalization sequence. Backfilling, in addition to exchange, is believed to be a key component in the ligation process, since the Raman EFs of PEG-MAuNPs functionalized with 4-MBA were markedly greater than (4-MBA)-MAuNPs not grafted with PEG. This could be due, in part, to PEG maintaining more completely dispersed particles that provide a greater surface area for 4-MBA functionalization. The samples all exhibited magnetic attraction to a permanent magnet, and were able to undergo magnetophoretic deposition. Overall, PEG-MAuNPs show promise as a SERS-based sensing platform for hydrophobic analytes. The ability of the nanoparticles to be magnetically deposited, and the simplicity of synthesis and modification procedures, are noteworthy.

## Figures and Tables

**Table 1:** Most probable pair distance of MAuNPs and various functionalized analogs, as determined by *P(r)* fits.

Sample	Most Probable Pair Distance (nm)
MAuNPs	8.8 ± 5.6
0.2 mM PEG-MAuNPs	8.3 ± 4.8
2 mM PEG-MAuNPs	7.4 ± 4.6
20 mM PEG-MAuNPs	9.9 ± 5.0
0.1 mM (4-MBA)-MAuNPs	8.8 ± 5.4
1 mM (4-MBA)-MAuNPs	8.2 ± 5.2
0.1 mM 4-MBA/2 mM PEG-MAuNPs	11.8 ± 5.4
2 mM PEG/0.1 mM (4-MBA)-MAuNPs	10.3 ± 5.0

**Table 2:** PEG and 4-MBA graft densities, as calculated by Equation (2). Error values shown were calculated using the standard deviation of the NMR data and the TEM D_MAuNP_ data.

Sample	PEG Graft Density (Chains nm^−2^ × 10²)	4-MBA Graft Density (Molecules nm^−2^)
PEG-MAuNPs	3.3 ± 3.1	0.4 ± 0.5
(4-MBA)-MAuNPs	0.1 ± 0.1	6.4 ± 5.8
PEG-(4-MBA)-MAuNPs	0.5 ± 0.4	7.0 ± 6.5
(4-MBA)-PEG-MAuNPs	4.7 ± 4.3	2.2 ± 2.0

**Table 3:** Power law fit parameter values with 95% confidence intervals (CI) and R^2^ values.

Sample	Prefactor, A (95% CI)	Power, b (95% CI)	R^2^
MAuNPs in DI Water	9.09 (9.05, 9.14) × 10⁻³	3.45 (3.44, 3.46) × 10⁻¹	1.000
MAuNPs in Chloroform	1.95 (−1.67, 2.06) × 10⁻²	0.43 (−1.02, 1.88) × 10⁰	0.966
(4-MBA)-MAuNPs in Chloroform (rinsed post-incubation)	0.69 (−0.36, 1.74) × 10⁻²	1.25 (−1.40, 3.90) × 10⁻¹	0.976
(4-MBA)-MAuNPs in Chloroform (direct from incubation solution)	0.36 (−1.40, 2.11) × 10⁻¹	0.96 (−7.87, 9.79) × 10⁻¹	0.685

## Data Availability

Not applicable.

## Supplementary Materials

The following supporting information can be downloaded at: https://www.mdpi.com/article/10.3390/nano12081253/s1, Figure S1: EDTA-MNPs, Figure S2: GISAXS, Figure S3: SEM, Figure S4: SERS of 4-MBA, PABA, and BA; Concentration Determination; NMR Sample Preparation.
