# Supplementary material for: Magnetic-Core/Gold-Shell Nanoparticles for the Detection of Hydrophobic Chemical Contaminants
Source: Nanomaterials (Basel). 2022 Apr 7;12(8):1253. doi: 10.3390/nano12081253 (PMC9027997; doi:10.3390/nano12081253)
Supplement: Supplementary file 1 [file nanomaterials-12-01253-s001.zip › nanomaterials-1621450-supplementary.pdf]

nanomaterials

Supporting Information

Magnetic-Core/Gold-Shell Nanoparticles for the Detection of Hydrophobic Chemical Contaminants

Anna M. Mills [1,2], Joseph Strzalka [3], Andrea Bernat [4], Qinchun Rao [4] and Daniel T. Hallinan Jr. [1,2,*]

[1] Chemical and Biomedical Engineering Department, Florida A&M University-Florida State University College of Engineering, Tallahassee, FL 32310, USA; amm15s@fsu.edu;
[2] Aero-propulsion, Mechatronics, and Energy Center, Florida State University, Tallahassee, FL 32310, USA
[3] Argonne National Laboratory, X-ray Science Division, Lemont, IL 60439, USA; strzalka@anl.gov
[4] Department of Nutrition and Integrative Physiology, Florida State University, Tallahassee, FL 32306, USA; asb13v@my.fsu.edu (A.B.); qrao@fsu.edu (Q.R.)
* Correspondence: dhallinan@eng.famu.fsu.edu; Tel.: +1-850-645-0131

(a)

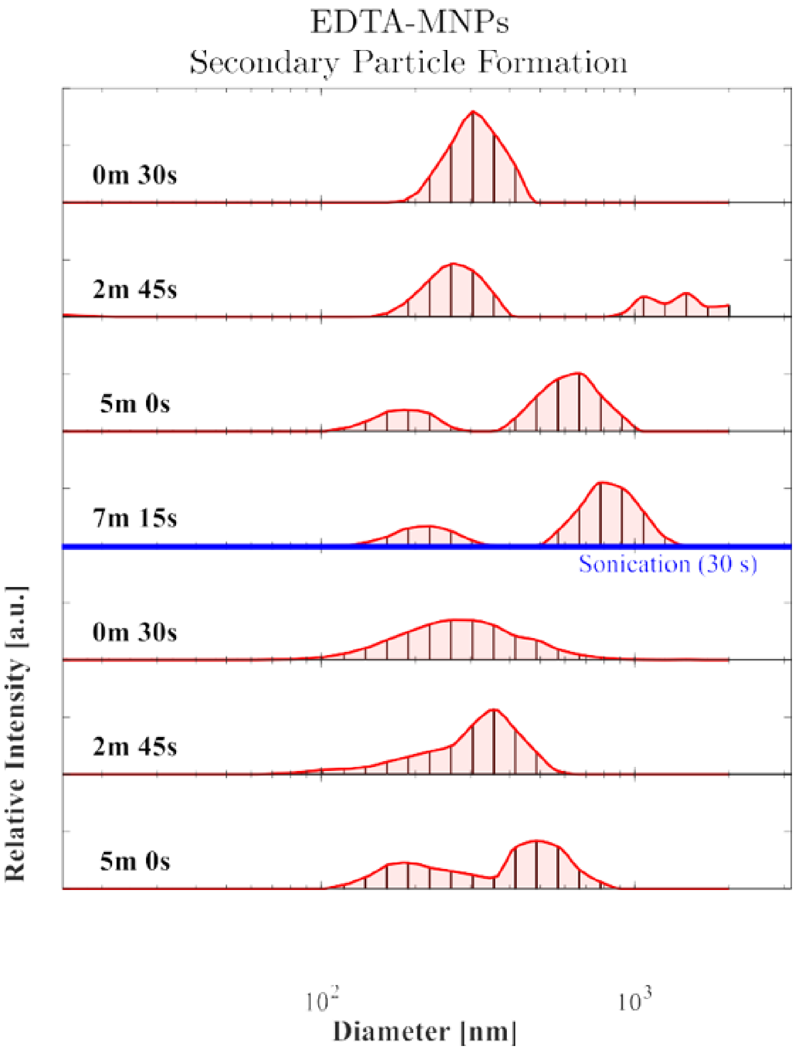

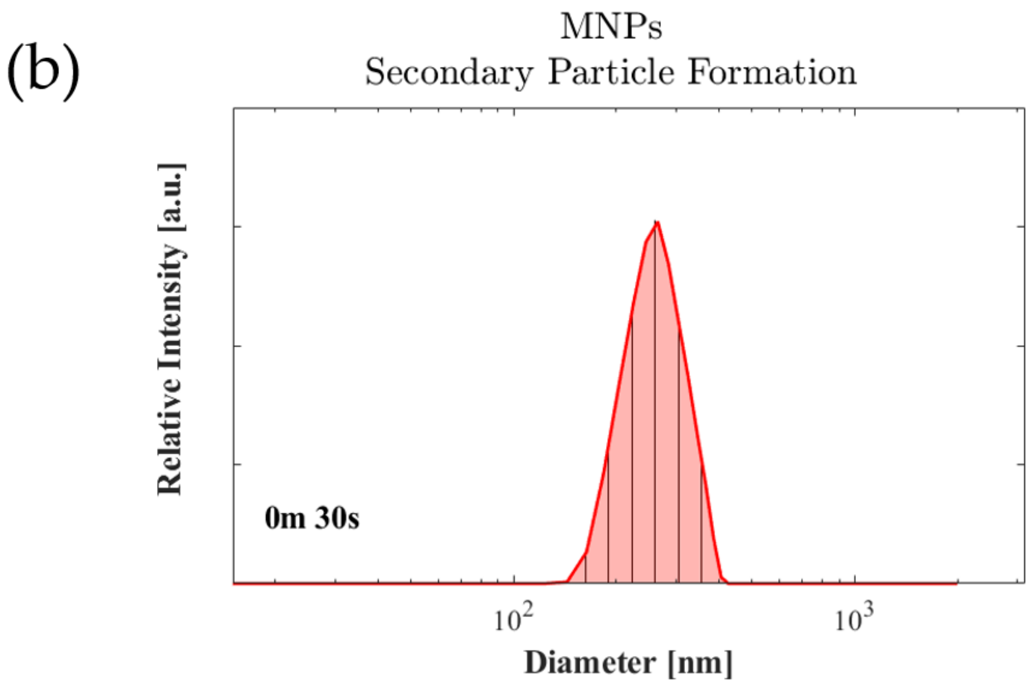

Figure S1. (**a**) Secondary particles begin to form very soon after sonication, but additional sonication re-disperses the particles and breaks up agglomerated EDTA-MNPs. (**b**) UncappedMNPs appear larger than what is observed in TEM.

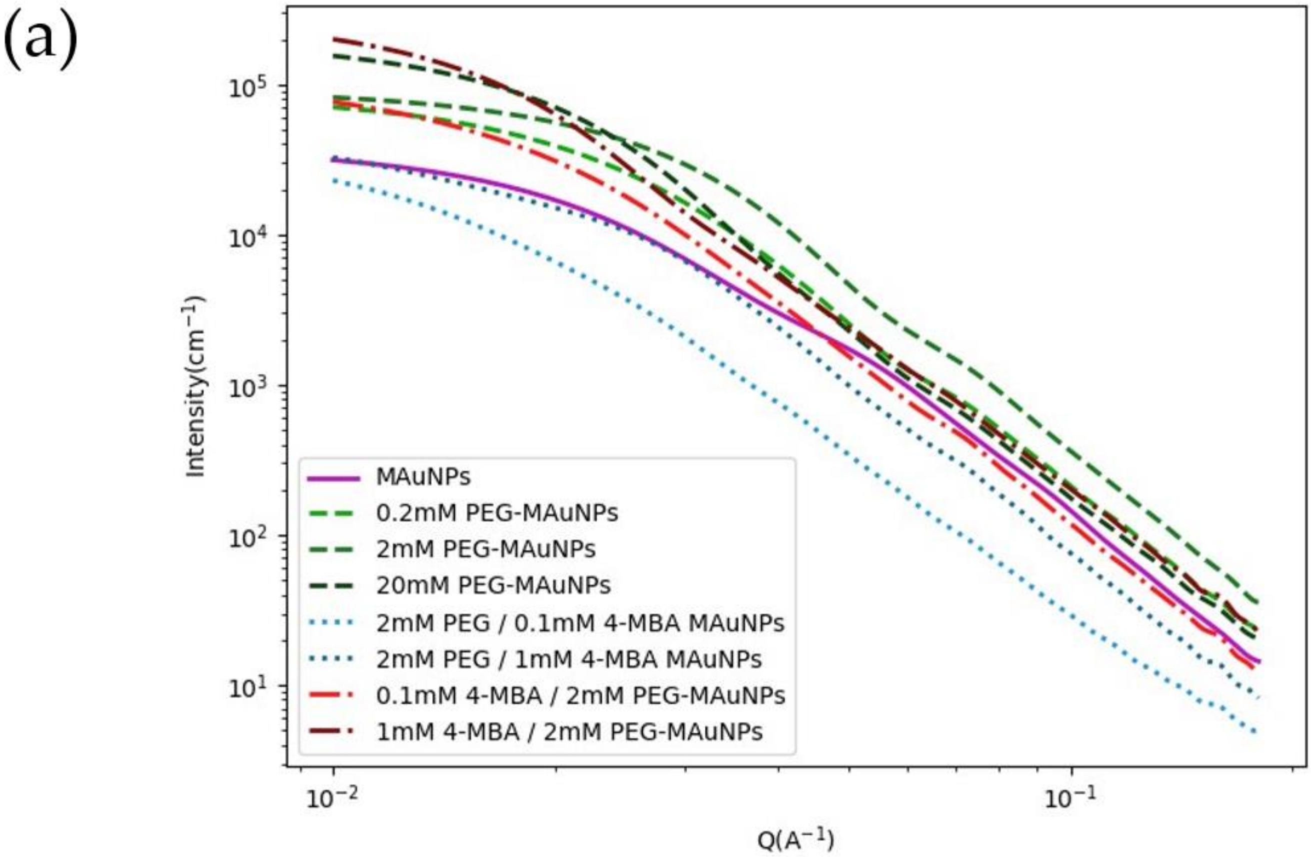

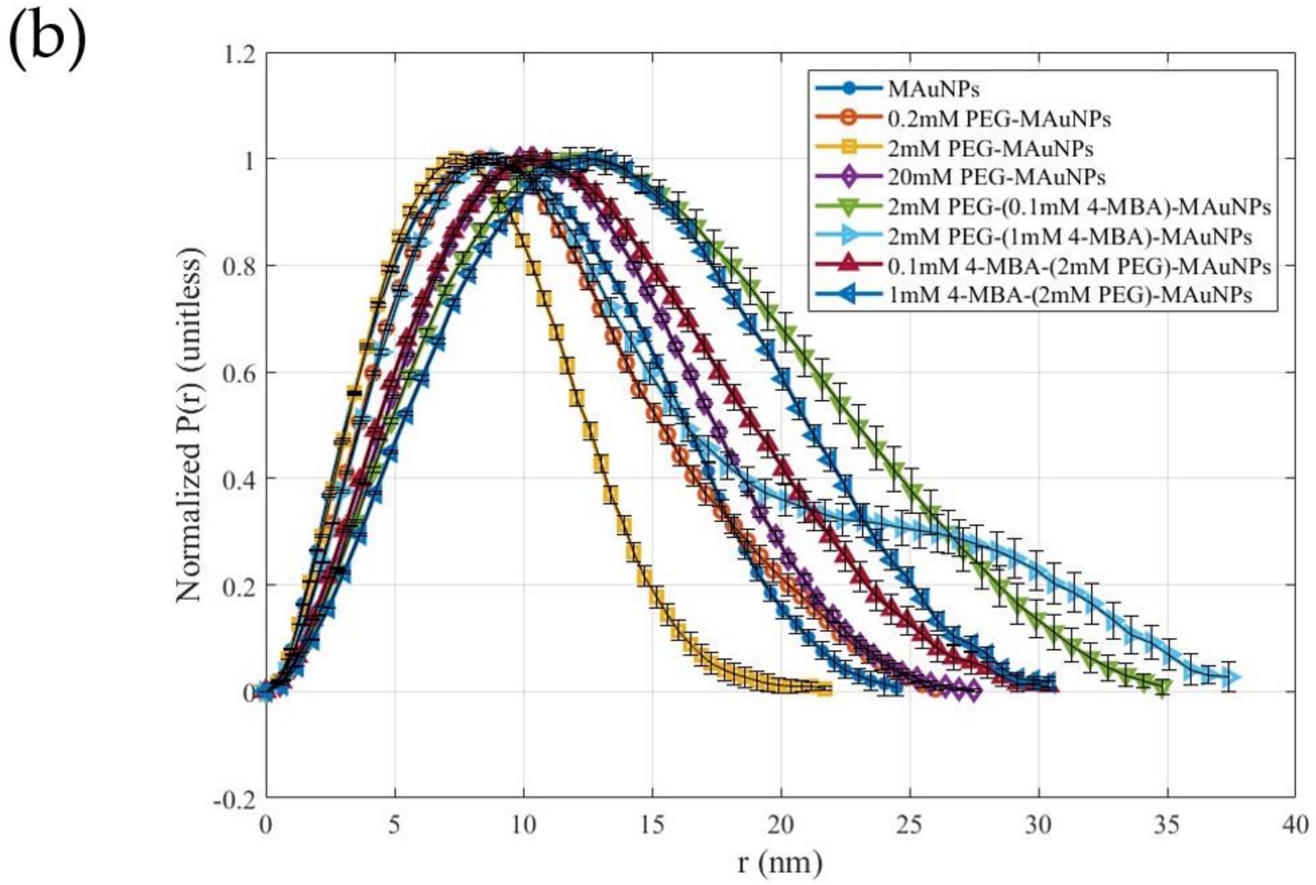

Figure S2. (**a**) Raw 1-D scattering data used to determine P(r). (**b**) Simulated P(r) fits weredetermined using SASView and normalized to a peak value of 1.

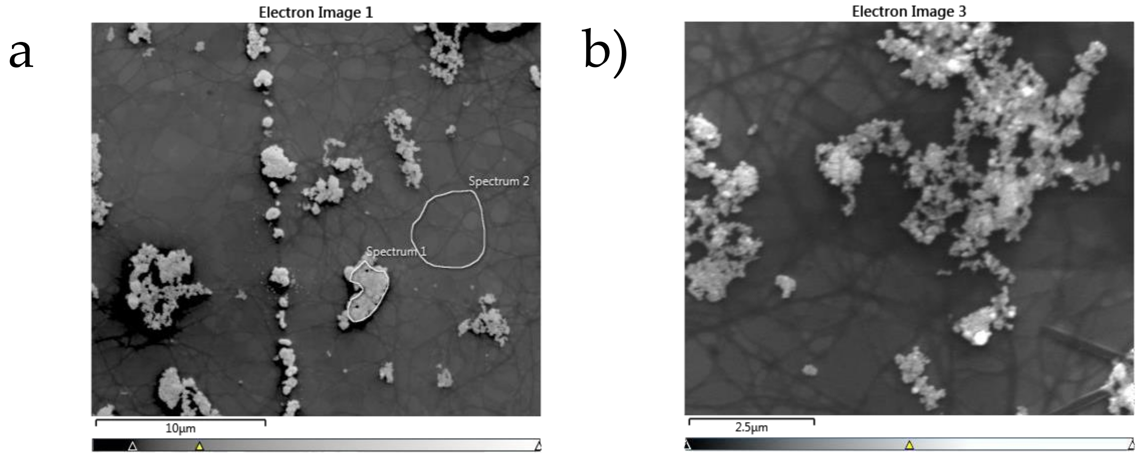

Figure S3. SEM micrographs of drop-cast (**a**) MAuNPs and (**b**) 0.2 mM PEG-MAuNPs.

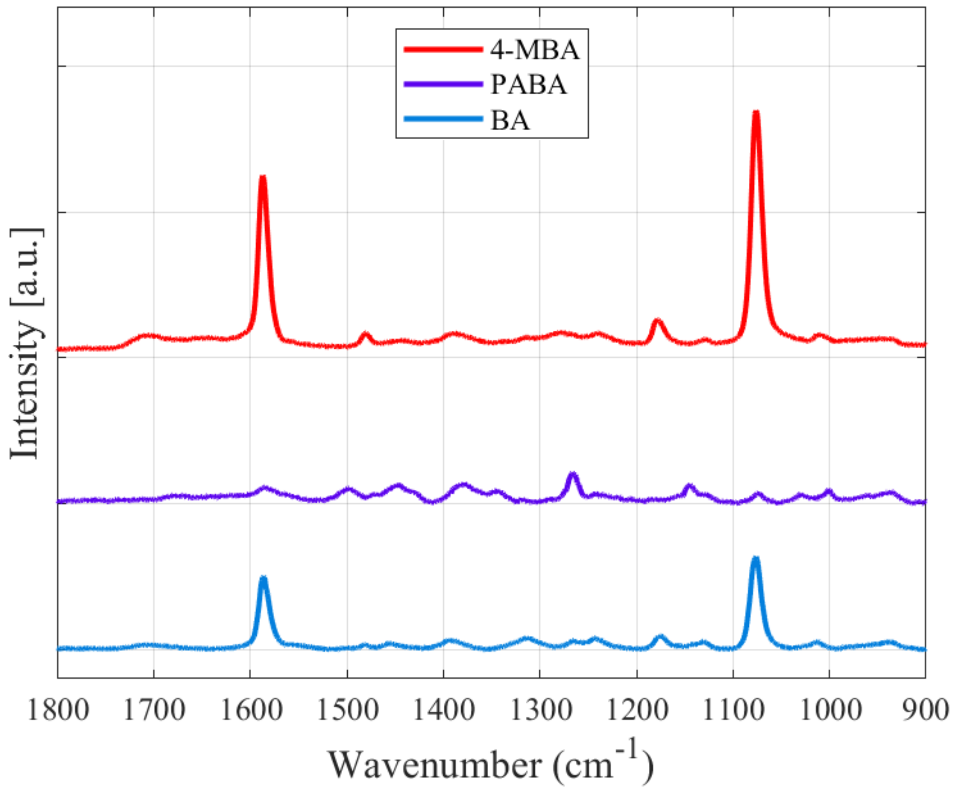

Figure S4. MAuNPs can be used to detect 4-MBA, PABA, and BA.

Concentration Determination

An aliquot of 1 vol% of the as-synthesized MAuNP solution was diluted to 0.1 vol% or 0.01 vol%. UV-vis was performed using 1 cm cuvettes, and the peak absorbance was recorded.Molar concentration was then calculated using the Beer-Lambert law (Equation (S1))

$$A = \varepsilon L c \quad \text{(S1)}$$

where A is the absorbance, ε is the molar attenuation coefficient, L is the path length, and c is themolar concentration. L was taken to be 1 cm, and ε was taken to be 3.36×10^9 $M^{-1}cm^{-1}$.[1]

Theoretical Mass Concentration Calculation

Using the bulk densities of Au and Fe_2O_3 and the nanoparticle dimensions as determinedby TEM, the theoretical mass concentration was determined.

$$\rho_{Fe_2O_3} = 5.24\ \mathrm{g/cm^3}$$

$$\rho_{Au} = 19.3\ \mathrm{g/cm^3}$$

$$D_{MNP} = 10.8\ \mathrm{nm}$$

$$D_{MAuNP} = 27.2\ \mathrm{nm}$$

The nanoparticle number density, ρ_N, was calculated by multiplying the molar concentration, M, by Avogadro's number, N_A.

$$\rho_N = M \cdot N_A$$

The mass of the MNP, m_{MNP}, was calculated by assuming a spherical geometry.

$$m_{MNP} = \rho_{Fe_2O_3} \cdot \frac{4\pi}{3\left(\frac{D_{MNP}}{2}\right)^3}$$

The mass of the Au in the MAuNP, m_{AuNP}, was also calculated by assuming a spherical geometry.

$$m_{AuNP} = \rho_{Au} \cdot \left(\frac{4\pi}{3\left(\frac{D_{MAuNP}}{2}\right)^3} - \frac{4\pi}{3\left(\frac{D_{MNP}}{2}\right)^3}\right)$$

The total mass of one MAuNP, m_{MAuNP}, was taken to be the sum of the masses of the core and theshell.

$$m_{MAuNP} = m_{MNP} + m_{AuNP}$$

Finally, the mass concentration of the nanoparticle dispersion, c_{MAuNPs}, was calculated with thefollowing equation.

$$c_{MAuNPs} = m_{MAuNP} \cdot \rho_N$$

NMR Sample Preparation

PEG-MAuNPs were synthesized, and 500 μL of concentrated PEG-MAuNP dispersion was collected. This dispersion was split evenly between a centrifuge tube and a microcentrifuge tube. The sample in the centrifuge tube was centrifugally rinsed three times with 3 mL D_2O (3,200 rpm for 5 min). 100 μL of the solution was collected and added with 390 μL D_2O and 10 μL 0.124 M pyridine in a 5 mm NMR tube. The sample in the microcentrifuge tube was dried under air, added with 100 μL chloroform and 1 mL 0.1 mM 4-MBA and sonicated for 30 s to re- disperse the PEG-MAuNPs in the incubation solution. After the 24-hour incubation period, the sample was centrifugally rinsed three times with 3 mL chloroform (3,200 rpm for 5 min). 100 μLPEG-(4-MBA)-MAuNP dispersion was extracted and subsequently dried under air. The sample was then re-dispersed in 100 μL D_2O using 30 s sonication. The sample was then added with 470μL D_2O and 10 μL 0.124 M pyridine in an NMR tube.

(4-MBA)-MAuNPs were prepared in 0.1 mM 4-MBA and rinsed with chloroform as described previously. 180 μL concentrated dispersion was extracted and split evenly between two microcentrifuge tubes. The sample in the first microcentrifuge tube was dried in air, re- dispersed in 2 mL D_2O (via 30 s sonication), and centrifugally rinsed three times with D_2O (3,200 rpm for 10 min). 35 μL concentrated (4-MBA)-MAuNP dispersion was extracted and added with 455 μL D_2O and 10 μL 0.124 M pyridine in an NMR tube. The sample in the secondmicrocentrifuge tube was dried in air, re-dispersed in 90 μL DI water (via 30 s sonication), and centrifugally rinsed three times with DI water. 20 μL concentrated (4-MBA)-MAuNP dispersionwas extracted and added with 2 mL of 2 mM PEG. This solution was left stirring overnight. Thesample was then rinsed centrifugally three times with 3 mL D_2O (3,200 rpm for 5 min). 60 μL concentrated (4-MBA)-PEG-MAuNP dispersion was extracted and added with 430 μL D_2O and 10 μL 0.124 M pyridine in an NMR tube.

Supporting Information References

1. Liu, X.; Atwater, M.; Wang, J.; Huo, Q., Extinction coefficient of gold nanoparticles with different sizes and different capping ligands. *Colloids Surf. B Biointerfaces* **2007,** *58*, 3-7.
